# Insights into the HIV Latency and the Role of Cytokines

**DOI:** 10.3390/pathogens8030137

**Published:** 2019-09-04

**Authors:** Joseph Hokello, Adhikarimayum Lakhikumar Sharma, Manjari Dimri, Mudit Tyagi

**Affiliations:** 1 Department of Basic Science, Kampala International University-Western Campus, Faculty of Science and Technology, Bushenyi, Uganda; 2 Center for Translational Medicine, Thomas Jefferson University, 1020 Locust Street, Philadelphia, PA 19107, USA; 3 Department of Biochemistry and Molecular Medicine, The George Washington University, Washington, DC 20037, USA

**Keywords:** HIV-1, latency, eradication, transforming growth factor-beta (TGF-β), resting memory CD4+ T-cells

## Abstract

Human immunodeficiency virus-1 (HIV-1) has the ability to infect latently at the level of individual CD4+ cells. Latent HIV-1 proviruses are transcriptionally silent and immunologically inert, but are still capable of reactivating productive lytic infection following cellular activation. These latent viruses are the main obstacle in the eradication of HIV-1, because current HIV-1 treatment regimens are ineffective against them. Normal immunological response against an antigen activates CD4+ naïve T cells. The activated CD4+ naïve T cells undergo cell cycle, resulting in further transformation and profound proliferation to form effector CD4+ T-cells. Notably, in HIV-1 infected individuals, some of the effector CD4+ T cells get infected with HIV-1. Upon fulfillment of their effector functions, almost all activated CD4+ T cells are committed to apoptosis or programmed cell death, but a miniscule fraction revert to quiescence and become resting memory CD4+ T cells to mediate a rapid immunological response against the same antigen in the future. However, due to the quiescent nature of the resting memory T cells, the integrated HIV-1 becomes transcriptionally silent and acquires a latent phenotype. Following re-exposure to the same antigen, memory cells and integrated HIV-1 are stimulated. The reactivated latent HIV provirus subsequently proceeds through its life cycle and eventually leads to the production of new viral progeny. Recently, many strategies against HIV-1 latency have been developed and some of them have even matured to the clinical level, but none can yet effectively eliminate the latent HIV reservoir, which remains a barrier to HIV-1 cure. Therefore, alternative strategies to eradicate latent HIV need to be considered. This review provides vital knowledge on HIV latency and on strategies to supplement highly active anti-retroviral therapy (HAART) with cytokine-mediated therapeutics for dislodging the latent HIV reservoirs in order to open up new avenues for curing HIV.

## 1. Introduction

Human immunodeficiency virus type-1 (HIV-1) infects human CD4+ T cells [1,2] and macrophages [3] via mucosal or blood contacts. The virus is then carried into the lymph nodes where it subsequently spreads into other lymphoid organs followed by enhanced virus replication and systemic infection [4]. After three to six weeks of primary infection, there is an onset of acute phase which is characterized by mononucleosis-like syndromes; fever, sores in mouth, pharyngitis, rash, myalgia, malaise, lymphadenopathy, headache, nausea and vomiting, lethargy, ulcers on the genitals, enlarged liver, weight loss, night sweats, diarrhea, and other neurological symptoms with a sharp increase in viremia in peripheral circulation [1]. The increase in viremia during the acute phase is also marked by a concomitant decline in the CD4+ T-cell population attributable to direct virus-mediated cytotoxicity or infection-induced cytotoxic T-cells (CTL)-mediated killing of virus infected cells [1,2]. Usually, the viremia peak resolves following HIV-1-specific immune responses, but this immunological response to infection is insufficient to completely suppress HIV-1 replication [1,2].

The acute phase of HIV-1 infection is followed by a chronic asymptomatic phase, referred to as “clinical latency”, which lasts for several years [2]. During clinical latency, HIV-1 replication kinetics are highly dynamic and characterized by gradual depletion of peripheral blood CD4+ T-cells [3]. In this stage of HIV infection, the virus continues to replicate at very low levels. Pantaleo et al. demonstrated that even though the viremia is low or undetectable in peripheral circulation, virus replication is enhanced in lymphoid organs, perhaps due to a spectrum of mechanisms such as viral accumulation, cellular activation, rapid viral turnover etc. [4,5,6]. In addition to direct virus-mediated cytotoxicity, infection-induced CTL-mediated killing of HIV-1 infected cells is a potential mechanism for T-cell depletion [1,2,7]. It is also believed that HIV-1 infection induces an autoimmune phenomenon throughout the course of infection, which causes hyper-activation of cellular immune response that results in non-specific killing of immune cells [2].

Progressive decline in host immunity during a prolonged period of the clinical latency phase results in the inability of the host immune system to respond to other invading pathogens and is referred to as acquired immunodeficiency syndrome (AIDS). This phase is marked by depletion of CD4+ T-cells, which is inversely proportional to virus load in peripheral circulation and lymphoid organs [7]. The inability of the host to activate an immunological response during the AIDS phase leads to an onset of a broad range of opportunistic infections associated with HIV-1 infection referred to as AIDS-defining illnesses [1,2,7]. Common co-infections, which induce AIDS-defining illnesses in HIV patients, include herpes simplex virus type-1 (HSV-1), salmonella, candidiasis, and toxoplasmosis. Under the normal immune system, these co-infections remain latent. However, due to deterioration of the immune system, these pathogens become reactivated and extremely difficult to treat and clear. Moreover, opportunistic infections, such as Kaposi’s sarcoma (KS)-associated herpesvirus (KSHV), hepatitis, M. tuberculosis, and P. carinii exhibit multiple strains, which further complicate infection dynamics and treatment outcomes. Earlier occurrence of these infections due to the lack of quick, effective, reliable, and affordable diagnostic tools results in delayed detection and treatment initiation. However, following the introduction of highly active antiretroviral therapy (HAART), AIDS-defining illnesses have reduced to negligible levels. Although HAART has significantly increased the lifespan of infected individuals, it is unable to eliminate HIV-1.

Toxicities arising from HAART, as well as from the chemotherapy, used in the management of malignancies, such as HIV-associated KS, further enhances the hardship of HIV patients. Additionally, the replication of the HIV-1 in immune-privileged sites with limited access to therapeutic drugs, and the ability of the virus to establish latent infection are the two main factors that hinder the eradication of HIV. The lack of a suitable animal model to study and evaluate novel therapeutic approaches, further hampers the discovery of novel therapeutic avenues. This review discusses in-depth details on latently infected HIV and possible strategies for supplementing anti-HIV therapy to dislodge HIV-1 reservoirs.

## 2. Establishment of HIV-1 Latency in Resting Memory CD4+ T-Cells

Upon pathogenic invasion, the quiescent naïve CD4+ T cells get activated after encountering the antigens presented by antigen presenting cells (APCs). Following antigenic-stimulation, naïve CD4+ T cells become metabolically active, undergo rapid multiplication, and transform into effector CD4+ T-cells. After clearing off the antigen from the system, most of these cells are destined to undergo apoptosis. However, a tiny fraction of these cells become resting memory CD4+ T-cells, which persist for a very long time, even for life. These memory cells are highly antigen-specific and vital to preserving the immunologic memory of the immune system, which permits the mounting of a quick immune response following a subsequent encounter with the same antigen [8].

**Figure 1:**
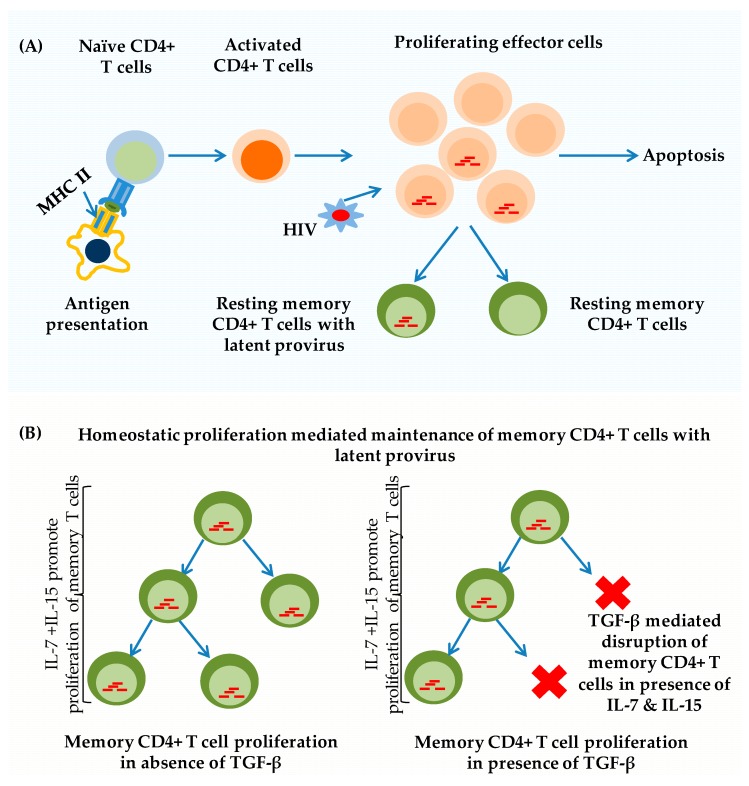
Mechanism of antigen presentation and the generation of latently-infected memory CD4+ T-cells. (**A**) Naïve CD4+ T-cells encounter antigen, become activated, and undergo enormous proliferation and differentiation to become effector CD4+ T-cells. Upon clearance of the antigen, many of these effector T-cells die, but a small fraction of the activated effector T-cells survive and revert back into quiescence. HIV-1 preferentially infects activated T-cells, and HIV latency is established when activated T-cells become infected and revert back to become memory T cell. Due to their quiescent nature, these cells are resistant to HIV superinfections, but are capable of reactivating productive infection following cellular activation. (**B**) Mechanism for the maintenance of HIV-1 latency in resting memory CD4+ T-cells under antiretroviral therapy (ART) and the potential effects of TGF-β on resting memory CD4+ T-cell proliferation. In the absence of TGF-β, latently-infected memory CD4+ T-cells are able to survive and proliferate periodically to replenish the latent HIV-1 provirus pools in presence of IL-7 & IL-15, Left panel. However, higher levels of TGF-β are able to disrupt the homeostatic proliferation of latently-infected resting memory CD4+ T-cells and restrict their number, Right panel.

Unlike naïve CD4+ T-cells, which are quiescent in nature and are unable to support productive infection, and the same as for efficient HIV-1 infection, metabolically active CD4+ T cells are required [9]. Following infection, a large number of infecting virions undergo functional decay, especially before or during the reverse transcription process [10]. HIV-1-mediated cytotoxicity further shortens the life span of effector CD4+ T-cells and thus reduces the efficacy of the immune system in HIV-1 infected individuals. In contrast, in memory T-cells, due to their quiescent nature, HIV-1 is unable to proceed through its life cycle and thus, it does not induce toxicity in memory T-cells. However, the long life span of the memory CD4+ T-cells, a necessity to maintain immunologic memory, allows the perpetual presence of transcriptionally-silent, latent HIV-1 proviruses in memory CD4+ T-cells [9,11] (Figure 1A).

Besides being metabolically inactive, several other factors impede infection of naïve or memory CD4+ T-cells by HIV-1. For instance, the surface of naïve CD4+ T-cells express very low levels of CCR5, an HIV-1 co-receptor, which results in the restricted infection by R5-trophic viruses. R5-trophic viruses constitute the vast majority of transmitting viruses during acute phase of primary HIV-1 infection. Furthermore, the presence of low molecular weight Apolipoprotein B mRNA editing enzyme 3G (APOBEC3G) in naïve CD4+ T-cells has been demonstrated to inhibit reverse transcription and HIV-1 infection of primary T-cells, coupled with limited availability of nucleotides in quiescent CD4+ T-cells [12,13,14].

Due to the quiescent nature, HIV-1 infection of naïve CD4+ T-cells is highly inefficient, and rarely proceeds to proviral integration into the host cell genome [15]. Persistence of HIV-1 DNA within activated CD4+ T-cells occurs in two forms, namely, a highly labile unintegrated form referred to as pre-integration latency, which decays within three months following initiation of HAART [16]. The second form, referred to as post-integration latency, is mediated by the presence of an extremely stable integrated HIV-1 DNA within the host chromosome, which persists forever despite prolonged and intensive HAART [17]. Post-integration latency represents a stable source of viral reservoirs in the resting memory CD4+ T-cell population, which reactivates productive systemic infection following cessation or disruption of HAART [17]. Besides those factors that restrict efficient HIV transcription, numerous other intrinsic factors of memory CD4+ T-cells that are detailed below play a role in restricting the reactivation of HIV-1, especially blockers of cellular innate immune responses [18,19].

Extensive studies, performed to analyze and characterize the behavior of latent HIV-1 proviruses in resting memory CD4+ T-cells isolated from peripheral circulation of patients on prolonged HAART, demonstrated that a very small fraction (100 per 10^6^ or 0.01%) of resting memory CD4+ T-cells actually harbor latent HIV-1 proviruses [18,19]. However, only 1 in 10^6^ of these latently-infected resting memory CD4+ T-cells are capable of reactivating productive HIV-1 infection upon stimulation [16,20,21].

## 3. Factors Mediating HIV-1 Latency in Memory CD4+ T-Cells

Unlike HIV-1 infection of activated CD4+ T-cells, which is characterized by high levels of virus replication, resting memory CD4+ T-cells support only restricted transcription of latent provirus [21,22]. Analysis of HIV-1 transcription patterns in latently-infected resting memory CD4+ T-cells has revealed the basal expression of short viral mRNA transcripts, with extremely low expression of complete genomic HIV-1 mRNA transcripts, which are necessary to generate new viral particles [16,21,22]. Cellular factors regulate HIV-1 latency both by controlling HIV transcription and the metabolic state of the HIV-1-harboring cell. Thus, latency is the result of multiple factors acting in concert, including (a) absence of nuclear forms of cellular transcription activation factors and sub-threshold levels of viral Tat protein; (b) epigenetic silencing of the HIV-1 long terminal repeats (LTR); (c) transcription interference; (d) microRNA-mediated degradation of viral mRNA and/or impaired HIV-1 gene expression; and (e) physiological maintenance of the quiescent memory CD4+ T-cell.

### 3.1. Absence of Crucial Transcription Factors from the Nucleus, and Sub-Threshold Level of Tat Proteins

Due to the intricate interdependence of viral LTR activation to cellular transcription factors and viral Tat, HIV-1 replicates potently in activated CD4+ T-cells unlike in latently infected memory CD4+ T-cells. Transcription factors that are critical for HIV transcription, such as nuclear factor kappa beta (NF-ĸB) and nuclear factor of activated T-cells (NFAT), are sequestered in the cytoplasm, and they only translocate to the nucleus following T-cell stimulation. Various T-cell stimuli, such as T-cell receptor (TCR) activation [23,24,25], phorbol esters induction [23,25,26,27] or cytokine stimulation [15,28], all promote nuclear translocation of NF-ĸB and NFAT and augment HIV transcription.

Due to restricted HIV-1 transcription, latently-infected memory CD4+ T-cells contain sub-threshold levels of viral Tat protein. However, T-cell activation increases the nuclear-translocation of NF-ĸB, which augments HIV-1 transcription, and consequently the production of viral proteins, including Tat. Once accumulated beyond the functional-threshold, Tat exponentially enhances HIV-1 transcription, after binding to the Trans activation response (TAR) element in nascent mRNA. The TAR is an RNA stem-loop structure present at the 5’ end of all viral transcripts. Tat brings Positive Transcription Elongation Factor-b (P-TEFb), a cellular transcription elongation factor, to TAR [29]. The cyclin dependent kinase-9 (CDK9) subunit of P-TEFb catalyzes the phosphorylation of several proteins at gene promoters, including phosphorylation of the c-terminal domain (CTD) of the largest subunit of RNA polymerase II (RNAP II), resulting in enhanced HIV-1 transcriptional elongation [13,29]. Thus, the binding of the Tat-P-TEFb complex to TAR increases HIV-1 transcriptional elongation efficiency by more than 100-fold, resulting in the generation of a large number of unspliced complete HIV-1 genomic transcripts [30,31,32]. We developed and used a novel model system to study HIV-1 latency, namely 2D10 cells, where HIV-1 Tat is expressed in cis and d2EGFP, a short half-life Green Fluorescent Protein (GFP), and replaces Nef to allow the shutdown kinetics of HIV-1 during entry to latency and reactivation from latency. Using this system [28], we observed that, indeed, reactivation of latent HIV-1 greatly depends on nuclear levels of NF-ĸB and viral Tat protein. Moreover, using a primary T cell based model for HIV latency, we have shown the restricted levels of P-TEFb in latently infected primary CD4+ T cells, and consequently, an additional block to HIV-1 transcriptional elongation during HIV latency in primary T cells [33].

### 3.2. Epigenetic Silencing of the HIV-1 LTR

Multiple cellular factors have been characterized to mediate HIV-1 latency. Initial reports indicated that cellular factors, such as yin yang-1 (YY1) and Late SV40 factors (LSF), cooperatively bind to the repressor complex sequence (RCS) within the HIV-1 LTR to mediate HIV promoter silencing through recruitment of histone deacetylase (HDAC) [34,35]. Williams et al. [36] subsequently reported that homodimers of NF-ĸ p50 binds to the HIV LTR to mediate HIV latency through recruitment of HDAC. Tyagi and Karn [37] showed that c-promoter binding factor-1 (CBF-1) mediates HIV-1 latency through recruitment of HDAC to the HIV-1 LTR in Hela and T-cells. In another set of experiments, Tyagi et al. [33] demonstrated that latent proviruses in primary CD4+ T-cells are enriched in heterochromatic markers, including HDACs and methylated histones. In microglial cells, which are the main target cells for HIV-1 infection in the central nervous system, Marban et al. [38], demonstrated that LTR silencing is mediated through recruitment of HADC1 and HDCA2 by co-repressor factor COUP-TF interacting protein-2 (CTIP2). Their group and others [39] demonstrated that histone methyltransferase SUV39H1 associated with CTIP2, and methylates histone H3 lysine 9 to promote HIV latency through formation of heterochromatic structure within the HIV-1 LTR. Similarly, Friedman et al. [40] demonstrated that HIV LTR silencing through an epigenetic mechanism was mediated through methylation of histones at the viral LTR by the histone lysine methyltransferase (HKMT) enhancer of Zeste-2 (EZH2). Pearson et al. further validated earlier findings and showed that HIV-1 latency entry is a step-wise phenomenon, where generation of transcriptionally-inhibitory chromatin structure is due to cumulative accumulation of numerous repressive epigenetic modifications at the viral LTR, plays a major role in inhibiting HIV-1 transcription during latency [28]. Numerous studies have shown that cellular factors play a key role in the establishment of the heterochromatin structures at LTR by recruiting different epigenetic enzymes, such as HDACs and HKMTs.

### 3.3. Transcription Interference (TI)

It was previously thought that HIV-1 latency in memory CD4+ T-cells was primarily due to the integration of HIV provirus within heterochromatin area of the host cell genome [41]. However, several reports have since demonstrated that the vast majority of latent HIV-1 proviruses preferentially integrate within actively transcribed cellular genes [20]. Transcriptional interference by the neighboring cellular promoters to the gene expression from integrated HIV provirus have also been shown to hinder HIV transcription and promote of HIV-1 latency. Upstream cellular promotor impede the HIV transcription by disrupting the formation or assembly of transcription complexes at the downstream HIV-1 LTR promoter finally resulting in the interruption of proviral transcription and HIV-1 replication [42]. Transcriptional interference has also been shown to occur by the neighboring cellular promoter, which is oriented opposite to HIV LTR promoter in the genome [43].

### 3.4. MicroRNA-Mediated Degradation of Viral MRNA and/or Impaired HIV-1 Gene Expression

Several reports have demonstrated that cellular microRNAs are differentially unregulated in resting memory CD4+ T-cells and mediate HIV transcriptional silencing and consequently, latency [44,45]. Usually, microRNAs (miRNA) impede translation after binding to the 3’ end of the mRNA, however, in certain instances, miRNA also results in mRNA degradation. The role of numerous miRNA in restricting HIV gene expression and mRNA degradation has been documented. In addition, different components of the cellular machinery that mediate miRNA-induced gene silencing have also been implicated in HIV-1 gene silencing and in promoting HIV-1 latency [46,47].

Altogether, these observations demonstrate that HIV-1 latency in resting memory T-cells is regulated predominantly at the level of transcription by multiple but complementary mechanisms acting in concert. Transformation to the memory phenotype is concomitant with rapid loss of transcription factors, such as NF-ĸB, NFAT and P-TEFb, from the nucleus, which results in the reduced rate of HIV-1 transcription and establishment of a heterochromatin structure at HIV-1 LTR. Heterochromatin structures strengthen the inertness of the LTR promoter and further impair HIV transcription from the LTR promoter. Restricted HIV-1 transcription is followed by the steady decline in Tat protein levels, and once Tat levels fall below the functional-threshold, HIV-1 attains a latent state [28].

Furthermore, using clone 2D10 cells, we have validated that stochastic fluctuations in LTR activity occur within populations of latently-infected cells was mainly due to the fluctuations in basal NF-ĸB and viral Tat levels [28,48,49]. Once HIV-1 establishes latency, transcription interferences play a crucial role in preventing stochastic reactivation of latent proviruses, and thus facilitate the maintenance of HIV latency. Together, it can be clearly envisaged that HIV-1 latency is predominantly maintained at the level of transcription by multiple mechanisms and cellular factors differentially acting in concert.

### 3.5. Homeostatic Maintenance of T-Cell Quiescence

The lack of certain transcription factors in the quiescent T cells not only inhibits the HIV-1 transcription, but also results in the overall reduction of cellular transcription. The quiescent cells, such as resting CD4+ memory T-cells, primarily the central memory T cells (T_CM_) and transitional memory T cells (T_TM_), are the most prominent cellular reservoirs of latent proviruses [50,51,52]. These memory T-cells are part of a mixed group of long-lived cells that express high levels of CD45RO in humans and CD44 in mice [53,54]. The survival of CD4+ memory T-cells for extended periods of time is well-established, but the mechanism responsible for their maintenance is not completely understood [55]. Both in humans and mice, among others, memory cells usually include a mix of central, transitional, and effector memory T-cells. Central memory T-cells, analogous to the naïve T-cells, express lymph node (LN)-homing receptors CD62L (L-selectin) and CCR7; consequently, similar to the naïve T-cells, they circulate through the LN and spleen. Meanwhile, effector memory T-cells downregulate CD62L and express diverse surface receptors that allow the cells to pass through non-lymphoid tissues [54]. The number of memory T-cells increases with age and presumably reflects the way that naïve T-cells respond to self-antigens and environmental diversity.

In contrast to naïve CD4+ T-cells, resting memory CD4+ T-cells divide intermittently, approximately once every 2 to 3 weeks. The gradual increase in the number of memory T-cells indicates that the amount of cell division is offset by an equivalent amount of cell death [56]. Notably, resting memory CD4+ T cells do not come in contact with self-p/MHC molecules, but do depend on their contact with interleukin-7 (IL-7) and interleukin-15 (IL-15) in order to proliferate homeostatically, and survive [57,58,59]. These findings were also validated using mouse models, in which most of the memory T-cells data derive from studies on naturally-occurring MP cells. These cells seem to be nearly indistinguishable from the resting memory T-cells that occur in response to defined antigens. Central memory CD4+ T-cells, which are the major latent reservoir, recirculate between blood and the secondary lymphoid organs, entering LN by expressing high levels of CCR7 and CD62L [54]. T_CM_ also express high surface levels of CD127 and CD122, and expression of these receptors enables them to proliferate homeostatically and survive in the presence of normal levels of IL-7 and IL-15 [57].

The central memory T cells (T_CM_) differentiate to either effector memory (TEM) following antigen-mediated stimulation or to transitional memory T cells (TTM) upon homeostatic proliferation [13]. These events lead to either partial or full reactivation of T_CM_, and so to an integrated latent HIV-1 provirus. However, in the continuous presence of anti-HIV drugs, reactivated latent HIV are unable to infect other cells, and HIV-1 levels return to the controlled range. Notably, cytopathic effects of the virus and host immune responses cause a large number of the differentiated cells to die, but some of these differentiated cells return to the T_CM_ phenotype with a few new mutations, due to restricted viral replication. These observations indicate that continual replenishment occurs in the latent reservoir in resting memory CD4+ T cells, acquiring updated viral sequences from time to time [60,61]. A similar phenomenon was found to occur in the SIV studies, which showed that in resting CD4+ T cells, the viral replication rate defines the SIV DNA sequence turnover [62]. Therefore, any strategy that selectively counters the homeostatic proliferation of latently infected resting memory CD4+ T-cells may be therapeutically relevant for controlling latent reservoir and curing HIV.

## 4. Current Approaches to Eliminate Latent HIV Reservoirs

The findings from the current investigations, focusing on HIV-1 persistence have open up new avenues for a wide variety of potential therapeutic strategies [63,64,65,66,67,68]. One of the approaches that has undergone several clinical trials is known as ‘shock and kill.’ In this strategy, latency reversing agents (LRAs) or transactivator(s) are administered to patients on HAART to reactivate latent HIV-1 by stimulating the transcription of latent/silent provirus and subsequent prevention of new infections through HAART [69]. This strategy aims to limit exposure to LRAs until the latent reservoir falls to an extent that would allow HAART discontinuation without the risk of viral rebound. The most common LRAs include histone deacetylase (HDAC) inhibitors/antagonists and protein kinase-C (PKC) activators/agonists. The ‘shock and kill’ strategy entails activation of HIV-1 expression to allow latently-infected resting memory CD4+ T-cells to die from viral cytopathic effects and/or host cytolytic immune effectors, while controlling new infections via HAART [69].

Arguably, this strategy seems promising for a scalable solution to HIV-1 eradication. There have been over 15 completed clinical trials testing LRAs from distinct mechanistic classes [70,71]. However, only modest perturbation to the reservoir was observed to-date. On the other hand, ex vivo experiments using aviremic patient cells have demonstrated that viral reactivation, even when using potent regimens, occurs for only a minority of latently infected cells after a single administration of the LRAs [72,73,74]. The reactivation of latent HIV-1 by LRAs was primarily limited to the circulating latently infected T-cells. Unfortunately, a majority of latently-infected CD4+ T-cells continue to hide in certain anatomical sites, where LRA access was highly limited. The key to utilize LRAs as part of a curative method for HIV-1 is that LRAs should not only be able to reduce the size of the latent reservoir, but they should also be able to restrict the re-emergence of the virus upon HAART discontinuation. To eliminate activated virus-expressing CD4+ T-cells, cytotoxic T-lymphocytes (CTLs) may also need to be boosted. In acute infection, CTLs are important for controlling HIV-1 replication, but in chronic infection, the cytolytic capacity of CD8+ T-cells is impaired and not restored by HAART. HIV acquires and maintains resistance mutations to CTLs in long-lived memory CD4+ T-cells due to the pressure from the CTLs and the evolution of the virus sequence during replication [75]. Considering the limitations of latency-reversing interventions observed thus far, additional strategies to deplete latently-infected resting memory CD4+ T-cells are needed.

## 5. Cytokines Modulation of HIV Infection

Infection with HIV is known to result in dysregulation of the cytokines, which play an important role in modulating the homeostasis of the immune system. Infection with HIV induces the production of pro-inflammatory cytokines, including interleukins, interferons and chemokines. Some of these cytokines regulate homeostasis of the immune system as well as HIV replication. HIV infection also tends to increase the production of T helper type-2 (Th2) cytokines (Interleukin-4 (IL-4), Interleukin-10 (IL-10)), proinflamatory cytokines (Interleukin-1 (IL-1), Interleukin-6 (IL-6), Interleukin-18 (IL-18)), and TNF-α; and it tends to decrease the level of T helper type-1 (Th1) cytokines, including Interleukin-2 (IL-2) and anti-viral interferon-gamma (IFN-α) [67]. Osuji; et al. [76] have shown that, compared with uninfected individuals, HAART-naïve HIV-infected individuals have elevated levels of proinflamatory cytokines (TNF-α, IL-6), anti-inflammatory cytokines (IL-4, IL-10), and transforming growth factor-beta (TGF-β). The level of TNF-α and TGF-β remained significantly elevated even 12 months after the initiation of HAART. 

Furthermore, it has also been shown that the cytokines TNF-α, TNF-β, IL-1 and IL-6 stimulate HIV-1 replication in both T-cells and monocyte-derived macrophages (MDM). The cytokines IL-2, IL-7 and IL-15 are also reported to upregulate HIV-1 replication in T-cells (Table 1). Meanwhile, the cytokines IFN-α, IFN-β, and IL-16 have been shown to repress HIV-1 replication in T-cells and MDM, while IL-10 and IL-13 inhibit HIV-1 replication in MDM [67]. The beta-chemokines, such as macrophage inflammatory protein (MIP)-1 alpha, MIP-1beta and RNATES, are inhibitors of macrophage-trophic strains of HIV-1 whereas the alpha-chemokines, such as stromal-derived factor-1, inhibit infection by T-trophic strains of HIV-1 [67]. As the disease progresses, the cytokines TNF-α, IL-2 and IL-6 regulate and replenish the latent HIV reservoir [69]. Immunosuppressive and pro-inflammatory cytokines favor the HIV latency by inhibiting the viral replication [70].

## 6. The Role of TGF-β in the Modulation of Latent HIV Provirus Pools in Resting Memory CD4+ T-Cells

HIV-1 remains dormant in CD4+ T lymphocytes and forms a reservoir, which is controlled but not eliminated by HAART. Even though, the success of HAART in HIV/AIDS therapeutics has extended the life span of many infected individuals, there is still room for improvement. The common HAART regimen consists of Nucleoside Reverse Transcriptase Inhibitors (NRTIs), Non-Nucleoside Reverse Transcriptase Inhibitors (NNRTIs) to inhibit the activity of reverse transcriptase (RT), and protease inhibitors (PIs) to restrict virus replication and proliferations. New generation of HAART regimens also contain viral entry and integrase inhibitors, which inhibit the cellular entry and integration of the HIV provirus into the host cell genome. The primary focus of the current anti-HIV therapy is to inhibit HIV replication and transmission, rather than HIV-1 eradication or cure. Therefore, under the current scenario, the infected individuals need to rely on HAART for the rest of their life. Hence, to restrain HIV-1, the infected individuals must take HAART regularly. However, continuous use of HAART drugs inflicts mild to serious side effects both for the short and long term. Some side effects appear for the first couple of weeks while others are continuous for months or even years after starting HAART. Individuals are required to continue HAART despite any side effects because discontinuation will allow HIV-1 to multiply and damage the immune system.

On other hand, enhancement of the immune system (immunotherapy) in infected individuals is one of the new approaches that is being tested in HIV-1 infected individuals. This approaches aim to enhance the innate powers of the immune system to fight back against the infections and other diseases. Gamma-Chain (γC) receptor cytokines, including Interleukin-2 (IL-2) [77,78], Interleukin-7 (IL-7) [79,80,81,82], Interleukin-15 (IL-15) [83] and Interferon (IFN) [84], have all been tested in immunotherapeutic clinical trials to treat and control HIV infection. 

Activated T-cells produce IL-2, which in-turn stimulate the T-cells itself and natural killer (NK) cells to proliferate, differentiate and release other cytokines. Activated T-cells also stimulate B-cells to release antibodies which protect the host against invading microorganisms [81]. IL-2 has been extensively studied in phase I, phase II and phase III studies. Although the use of IL-2 demonstrated conflicting results in clinical trials [85], it still restored immune functions in some HIV-positive patients [77,78]. Studies have shown that intravenous or subcutaneous use of IL-2 can induce significant increases in CD4+ T-cells in HIV patients depending on the dose and when preferentially administered along with HAART [77,78,86]. However, finding from the ESPRIT Study Group and SILCAAT Scientific Committee indicated that supplementation of HAART with IL-2 offers no clinical benefit as compared with antiretroviral therapy alone.

The functions of (interleukin-7) IL-7 have also generated interest in its utilization to boost de novo T-cells formation following T-cells deficiency caused by HIV infection. IL-7 is known to be involved in mediating the survival of early thymocytes [87,88] and in promoting the survival of both naïve and memory T-cells [59]. Results of the INSPIRE 2 clinical trials using recombinant human IL-7 (r-hIL-7) showed that r-hIL-7 was well-tolerated and resulted in sustained restoration of CD4+ T-cells in the majority of HIV patients undergoing HAART [79]. Similar results were obtained in other studies utilizing r-hIL-7 [80,81,82]. In contrast, IL-7 use was reported to lead to rapid proliferation of the latent HIV reservoirs in resting memory CD4+ T-cells [89]. Nevertheless, Vandergeeten C et al. reported in 2013 that IL-7 promotes mechanisms of HIV-1 persistence during HAART by enhancing residual levels of viral production and inducing proliferation of latently infected cells [89]. Study further indicated that IL-7 does not represent a suitable candidate therapeutic strategy for HIV eradication.

Researchers have also explored the potential of IL-15, which is a pleiotropic cytokine with diverse biological functions and which plays a crucial role in host defense from viral and non-viral intracellular pathogens. Along with other γC cytokines, such as IL-2 and IL-7, IL-15 facilitates the maintenance of naïve and memory T-cell populations [59,90]. Most recently, ex-vivo experiments using IL-15 with samples obtained from HIV patients on HAART have demonstrated that IL-15 treatment improved NK-cell functions, but more importantly, IL-15-treated NK-cells were able to clear latently HIV-1-infected cells following exposure to Vorinostat, an LRA [83].

Interferons (IFN), on the other hand, are innate antiviral proteins known to restrict viral replication even before any antibodies are produced. Interferons are reported to mediate potent antiviral effects in resting memory CD4+ T-cells and other cell types through APOBEC3G [91]. For example, IFN-α is quite extensively used in the treatment of hepatitis B (HBV) and C (HCV) virus infections. Interferon-α is a product of a multigene family encoding 12 IFN-α subtypes [92] all of which bind to IFN-α/β receptors but with varied biological outcomes. With regards to IFN and HIV-1 immunotherapy, recent studies have demonstrated that all of the 12 IFN-α subtypes exhibit unique host responses and display distinct efficacies in the control of HIV-1 infections [84]. For instance, a recent in vivo study utilizing humanized mouse models has shown that IFN-α14 subtype but not IFN-α2 potently inhibits HIV-1 replication and suppressed HIV-1 load [84].

In consideration of the foregoing observations regarding the use of cytokines as immunotherapeutic agents in the treatment and control of HIV-1 infections, we hereby commented on a novel approach of utilizing Transforming Growth Factor- beta (TGF-β) to eradicate latent HIV-provirus pools in HIV patients receiving HAART. Transforming growth factor-beta is a pleiotropic cytokine that functions in numerous physiological and pathological processes. The TGF-β—including TGF-β1, TGF-β2 and TGF-β3 isoforms—is a 25kDa homodimeric cytokine. Most cell types express TGF-β gene, however, particular isoforms of TGF-β appear to be expressed based on tissue specificity. The TGF-β1 is the predominant isoform expressed by most immune cells. Members of the TGF-β family regulate multiple cellular functions, such as, proliferation, differentiation, and migration, with a range of other diverse biological activity [93,94], including inflammation, wound repair, and immune homeostasis and tolerance [95].

The activity of TGF-β is tightly regulated both positively and negatively, from the time of secretion to activation of the target genes. Active TGF-β is cleaved from a precursor protein, latent TGF-β binding protein (LTBP), which is stored in the extracellular matrix [96]. The activated TGF-β signals through the TGF-β receptor complexes and the serine/threonine kinases, namely TbetaRI and TbetaRII. Receptor activation results in the phosphorylation of several downstream targets, including smads [96,97]. Phosphorylated smads subsequently regulate (both positively and negatively) the expression of TGF-β target genes [97]. 

Research which showed that TGF-β supports wound repair by augmenting collagen synthesis was the first to identify its defined, non-transforming role [98,99,100,101]. Later research showed that activated lymphocytes also produce TGF-β, and that TGF-β potently suppresses lymphocyte proliferation [102,103,104,105]. TGF-β regulates HIV replication directly by acting on infected cells; however, TGF-β can either stimulate or inhibit HIV replication, unlike monophasic stimulation of HIV replication by cytokines (i.e., IL-1, TNF-α, GM-CSF, LIF) or monophasic inhibition in response to interferons. The doses and the cell system, along with the presence or absence of other cytokines, affects the quality and eventual impact of the TGF-β. For instance, Czubala et al. [106] reported that TGF-β induces the sterile alpha motif (SAM) and histidine aspartic (HD) domain protein-1 (SAMHD1)-independent post-entry restriction to HIV-1 infection in monocyte-derived langerhans’ cells and epithelial langerhans’ cells. In contrast, Theron et al. [107] recently observed that elevated circulating TGF-β contributes to immunosupression in both untreated and treated HIV-1 patients and progression to Acquired Immunodeficiency Syndrome (AIDS) in untreated HIV-1 patients. They further observed that TGF-β may be linked to the pathogenesis of non-AIDS-defining cardiovascular, hepatic, renal and pulmonary disorders. Despite these observations, we propose that TGF-β could be used transiently in HIV patients undergoing HAART in order to deplete the latent HIV reservoirs in resting memory CD4+ T-cells so as to allow discontinuation of HAART.

As discussed in the foregoing sections, one of the mechanisms to maintain HIV latency is the homeostatic maintenance of the resting memory phenotype. It so happens that these resting CD4+ T-cells harbor latently-infected HIV proviruses and these T-cells subset are capable of intermittent proliferation in order to replenish the latent HIV reservoirs [108]. In this regard, this review discussed a novel approach to disrupt and deplete the latent HIV reservoirs in resting memory CD4+ T-cells utilizing only transient use of TGF-β, which is shown to inhibit homeostatic proliferation of resting memory CD4+ T-cells. To this effect, several studies have shown that TGF-β potently inhibits homeostatic proliferation of resting memory CD4+ T-cells [109] (Figure 1B). Tiemessen et al. showed how TGF-β affects antigen-specific proliferation and the CD4+ T-cell activation status and cytokine production. They also showed that TGF-β adequately and potently suppresses antigen-specific resting memory CD4+ T-cell proliferation by cell-cycle inhibition rather than apoptosis induction. Moreover, on CD4+ T-cells, TGF-β increased CD69 expression and decreased CD25 expression, indicating that it can modulate activation of already-differentiated CD4+T-cells. Accordingly, Das and Levine [110] reported that TGF-β inhibits IL-2 production and subsequently blocks entry of memory CD4+ T-cells in to the cell cycle even in the presence of sustained T-cell receptor activation. Consistent with the observations of Das and Levine, Mckarns and Schwartz [111] demonstrated that TGF-β-mediated impairment of memory CD4+ T-cells entry and subsequent progression through the cell cycle. In a separate set of experiments, Mckarns et al. [112] further demonstrated that this inhibition was mediated via Smad3. Similarly, of particular importance, most recently, Nguyen and Sieg [113] vividly demonstrated that TGF-β inhibits IL-7-induced proliferation of resting memory CD4+ T-cells but not naïve T-cells. Interestingly, resting memory CD4+ T-cells are the same T-cells subset that harbors latent HIV proviruses and their homeostatic proliferation is induced by IL-7 and IL-15. These observations strongly suggest that utilization of TGF-β in combination with HAART could specifically disrupt IL-7-mediated homeostatic proliferation and replenishment of resting memory CD4+ T-cells so as to deplete the latent HIV reservoirs from peripheral circulation. It is worth mentioning that elevation of circulating TGF-β levels certainly blocks the proliferation of not only TCR-activated but also IL-7-induced resting memory CD4+ T cells. Together, these findings suggest the potential transient therapeutic use of TGF-β in controlling the reservoir size and combating HIV-1 proviral latency [109,110,113].

## 7. Conclusions

HIV-1 establishes latent infection in CD4+ memory T cells and persists indefinitely, even in individuals who are on HAART. The stability of latently-infected resting memory CD4+ T-cells is not, for the most part, due to new de novo infection events during HAART, but rather to the ability of resting memory CD4+ T-cells to proliferate and promote immunologic memory. Even though the current treatment regimens for HIV have greatly improved the life expectancy of HIV/AIDS patients, the current HAART therapies, which inhibits the replicating virus are unable to eliminate these latent infected viruses. The quality of life in HIV/AIDS patients on HAART is compromised since they need to adhere to it for rest of their lives to prevent the virus from rebounding. Long-term HAART causes drug toxicities, numerous side effects, and complications. Therefore, treatment options that target both the replicating and latently infected virus are the need of the hour. We believe that the application of adjuvant therapy in combination with HAART could prove more effective in eradicating not only the replicating virus but also the latent virus. This approach may disrupt and deplete the latent HIV reservoirs in resting memory CD4+ T-cells and thus potentially remove the need to rely on HAART for lifelong. The information generated from this review will provide vital information on HIV latency and the strategy of adjuvant immunotherapies for depleting latently-infected resting memory CD4+ T-cells in HIV patients. The success of these strategies will depend upon a greater understanding of the causative factors responsible for immune activation and immune responses.

## Figures and Tables

**Table 1:** List of Cytokines shown to modulate HIV replication.

Impact on HIV Replication	Cytokines
Enhance HIV replication in most of the cells	IL-1, IL-2, IL-4, IL-6, IL-7, IL-15, IL-18, TNF-α, TNF-β, M-CSF
Repress HIV replication in most of the cells	IL-10, IL-13, IL-16, IFN-α, IFN-β, SDF-1, MIP-1α, MIP-1β, RANTES
Enhance/reduce HIV replication depending on type of cells	IL-4, IL-12, IFN-γ, GM-CSF

IL: interleukin; SDF: stromal derived factors; MIP: macrophage inflammatory protein; TNF: tumor necrosis factors; INF: interferons; GM-CSF: Granulocyte-macrophage colony-stimulating factor.
